# Prostate Artery Embolization via Distal Transradial Artery Access in a 100-Year-Old Patient

**DOI:** 10.3390/jpm14010011

**Published:** 2023-12-21

**Authors:** Shauh-Der Yeh, Yu-Shiou Weng, Chun-Yu Lin

**Affiliations:** 1 Department of Urology, Prostate Center of Excellence and Cancer Center, Taipei Medical University Hospital, Taipei 11031, Taiwan; 2 Department of Urology, School of Medicine, College of Medicine, Taipei Medical University, Taipei 11031, Taiwan; 3 Department of Radiology, Tri-Service General Hospital, National Defense Medical Center, Taipei 11490, Taiwan; 4 Department of Radiology, Yonghe Cardinal Tien Hospital, New Taipei City 23445, Taiwan

**Keywords:** distal transradial (dTRA), PAE, BPH

## Abstract

Benign prostatic obstruction (BPH) is a common disease in males and surgical treatment is the gold standard for this symptomatic disease. Prostate artery embolization (PAE) is one of the emerging therapies which aims to minimize the lower urinary tract symptoms (LUTS) of BPH and the volume of enlarged prostates. We reported here a case of 100-year-old man with 90 cm^3^ prostate and severe symptoms secondary to BPH, who underwent a successful PAE through distal transradial access without any complications. The patient was satisfied with this treatment and no symptoms recurred after PAE. This demonstrated that PAE was a safe and effective treatment for BPH and was recommended for elderly/non-surgical candidates.

## 1. Introduction

Benign prostatic hyperplasia (BPH) is a common disease in males and surgical treatment is the gold standard for this symptomatic disease. Prostate artery embolization (PAE) is one of the emerging therapies which aims to minimize the lower urinary tract symptoms (LUTS) of BPH and the volume of enlarged prostates. Compared to surgery, PAE is a minimally invasive procedure and is performed under local anesthesia, which is beneficial to those high-risk BPH patients requiring surgical treatments. For example, in patients with a high risk of bleeding, general anesthesia is generally not suitable for them. For those with poor cardiac or lung functions, or extremely elderly patients, PAE has shown to have a lower incidence of severe complications. But, PAE can be a difficult procedure sometimes, due to the different types of origin of prostate arteries, combined with tortuous courses and stenosis. Recently, there was a 100-year-old man with good past health admitted for BPH treatment presenting refractory urinary tract infections (UTIs), recurrent gross hematuria, and nocturia three times every night. He had been diagnosed with BPH since 2013 and prior medical treatments including Tamsulosin, Dutasteride, and Bethanechol, etc., failed to control his BPH-related symptoms. His prostate volume was found to be 89.39 cm^3^. Surgical treatments were not considered, and he was referred to our department for PAE in September 2020, as he was committed to BPH treatment for a better quality of life and less frequent hospitalization for UTIs and hematuria.

## 2. Methods

**Figure 1:**
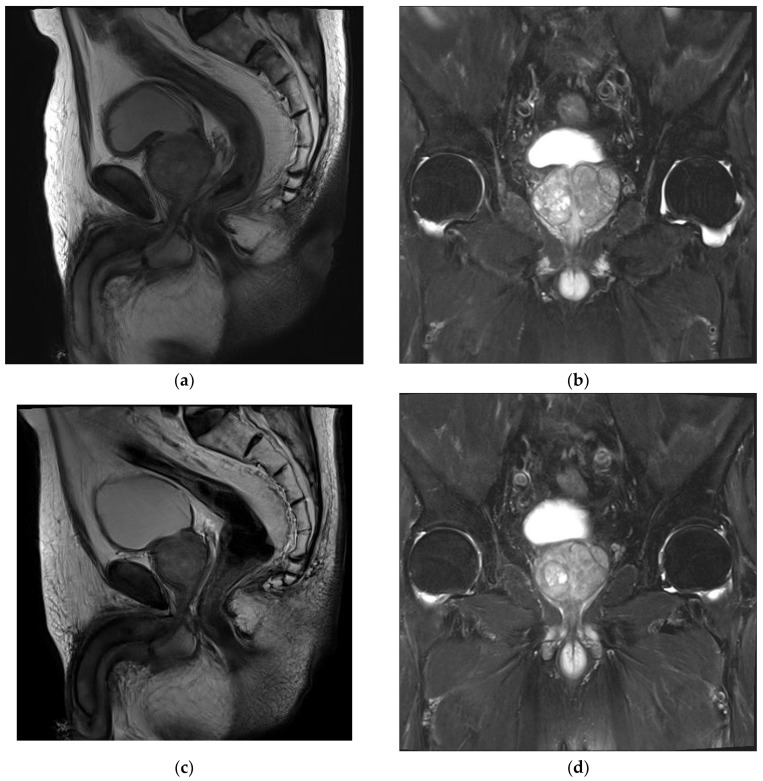
MRI images of patient’s BPH. (**a**,**b**) Pre-procedural MRI showed enlarged prostate of 89.39 cm^3^ (5.90 cm × 4.41 cm × 3.84 cm). (**c**,**d**) Post-procedural MRI at 24 months showed 66.05 cm^3^ (4.92 cm × 3.58 cm × 3.75 cm).

A pre-PAE MRI showed an enlarged prostate with a central gland of 89.39 cm^3^ (5.90 cm × 4.41 cm × 3.84 cm) (Figure 1a,b). No suspicious prostate-cancer-like lesion was identified. After discussion with urologist, this patient was identified as a PAE candidate.

**Figure 2:**
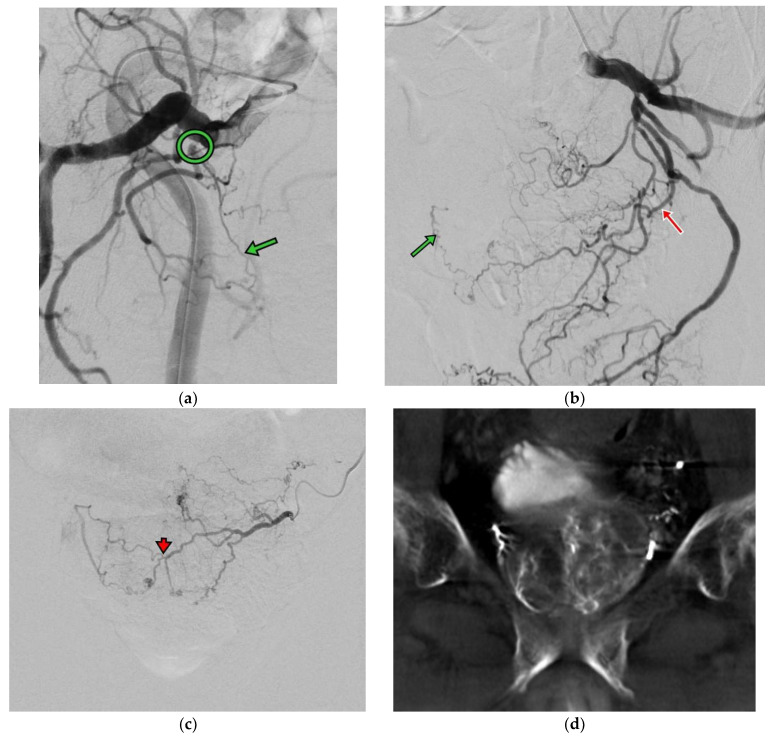
(**a**) Right internal iliac angiography (oblique view RAO 30 degrees) shows high grade stenosis (circle) at the branch of the right prostate artery (green arrow). (**b**) Left internal iliac angiography (oblique view LAO 30 degrees) shows the left prostate artery (red arrow) with contrast opacified at the right side of the prostate artery (green arrow). (**c**) Left prostate angiography (AP view) shows both sides’ prostate perfusion via intraprostatic anastomosis (arrowhead). (**d**) CBCT images show prostate perfusion at both sides on the left prostate angiography.

The PAE procedure was performed via left distal transradial access (dTRA). After local anesthesia (LA), 3000 IU heparin was injected via sheath. A 125 cm catheter (Ultimate, Merit Medical Inc., South Jordan, UT, USA) was introduced via distal radial artery straight down to the common iliac artery and was navigated to the right internal iliac artery. The procedure was complicated using severe stenosis in the anterior division of the right internal iliac artery, and a microcatheter advancement toward the right prostate artery was not feasible. Right femoral access was attempted but again failed due to stenosis (Figure 2a). Left prostate artery showed both sides’ prostate perfusion and was inaccessible to a 2.4 F microcatheter (150 cm Maestro, Merit Medical Inc.) through intraprostatic anastomosis (Figure 2b,c). A pre-filled syringe of 300–500 um Tri-acryl gelatin microsphere (Embosphere, Merit Medical Inc.) was mixed with 10 mL iodinated contrast agent, and 1 mL of the microsphere was injected slowly until the antegrade flow stopped. Bilateral PAE was performed by injection with microsphere from the left prostate artery. Procedural time was 220 min. Hemostasis by compression was performed for 2 h and bedrest was performed for 4 h. The patient was discharged on the next day without complications.

## 3. Results

The patient was followed up with after 1 week in the Department of Urology, and a significant improvement in his symptom score was noted. A further improvement was found along the follow-ups, with 78.3% at month 12 and a total improvement in IPSS of 18 points. The patient’s quality of life (QoL) was improved significantly from 5 to 1 at week 1 and good through the last follow-up. A six-month MRI showed a significant prostate volume reduction of 20.3% and 12- and 24-month MRIs with 28.6% and 35% reductions, respectively (Figure 1c,d). The PSA dropped 47.3% from baseline at month 12. The post-void residual volume improved by 43.5% at a 1-year follow-up from 155 mL at baseline to 87.6 mL. The patient stopped medical treatment at month 12 and no longer continued thereafter. No further hospitalizations occurred regarding BPH after PAE nor symptom recurrences for more than 3 years. The patient lived a normal life with minimal LUTS. Table 1 describes the clinical outcomes of this patient.

## 4. Discussion

Benign prostatic obstruction (BPH) is a common disease in males. Fifty percent of males aged 51 to 60 will have BPH, and the percentage will increase by age and by 75% at 70 years of age [1]. Although treatments for BPH have emerged in recent decades, surgical treatments are the gold standard for symptomatic BPH refractory to medical therapy [2]. However, patients older than 80 years of age are usually not suitable for surgeries and minimally invasive options must be explored. PAE has been proposed for many years as an alternative to surgery and has been recognized in many countries [3,4]. The indications of PAE in our institute include BPH-related LUTS management, and the advantages of PAE are minimally invasive under LA while maintaining the patient’s sexual function.

To our knowledge, this is the first case that presents the PAE treatment of a 100-year-old man via dTRA demonstrating its feasibility, safety and clinical efficacy with a 2-year follow-up. This patient had severe LUTS secondary to BPH since 2013, with an IPSS score of 23, was refractory to medical treatment and was a non-surgical candidate. PAE brought a new insight into symptomatic BPH management and, together with dTRA, the patient did not need to stop antiplatelet drugs with the easing of hemostasis, a shorter post-procedural bedrest, and an ambulatory procedure, which is demonstrated to be safe for elderly and non-surgical candidates [5,6]. This patient reminded us of the needs of bothersome LUTS’ management and a better quality of life.

Aligned with previous investigations, our report also showed significant LUTS improvement from Severe at baseline to Moderate at 1 month and further down to Mild at 6 months. There was a 78.3% improvement relevant to 18 IPSS points at 1 year and good improvement at 2 years. PAE was demonstrated to be effective in reducing the prostate volume by 35% without any complications [7]. Although only the unilateral prostate artery was selected with the microcatheter, on both sides PAE was conducted for this case using a cone beam CT(CBCT) image to confirm. This is the first reported image to show a prostate perfusion on both sides of a unilateral prostate artery due to severe stenosis on the other side. Although unilateral PAE was performed for this case, which was shown to be one of the negative factors for clinical success, the clinical outcomes were found to be comparable to the existing reports [8]. In Bilhim et al., 2013, a bilateral PAE led to better clinical results, but 50% of patients who received unilateral PAE also showed a good clinical outcome, like in this case [8]. From our experience, unilateral PAE is considered a technical success, and an improvement of IPSS by 25% or more from baseline is determined as a clinical success. In this case, both technical and clinical successes were achieved, and PAE was shown to be safe and effective for elderly patients who are eager to treat the bothersome LUTS secondary to BPH. No symptom recurrence after PAE was identified.

The lesson learned from this PAE case is procedural time management. A prior pelvic CTA must be performed to identify the vessels and stenosis for better PAE planning and a potential saving in procedural time. The technical aspects are still the main hurdles due to the anatomical variants of prostate arteries and arterial stenosis. CTA is suggested to map all vessels for PAE. To promote PAE as a treatment option for BPH in Taiwan, standardized measurements of clinical outcomes from PAE have to be implemented in our institutes including IIEF, QoL and Urodynamics. A technical guide for PAE via dTRA must be established. dTRA could be the first choice of access if the patient’s radial artery is larger than 2.6 mm with the use of a radial sheath or, if larger than 1.6 mm, with the use of a thin-walled sheath. It is now spreading in many institutes for outpatient/day procedures [9]. However, due to the limitation of catheter availability in Taiwan, for BPH patients, who are fit for PAE and taller than 175 cm, dTRA is not feasible and transfemoral access will be chosen. Currently, in Taiwan, the longest diagnostic catheter is 125 cm and sometimes it cannot reach the internal iliac artery. In this case, only the microcatheter can go through the internal iliac artery, which could make angiography difficult for identifying the prostate arteries solely based on angiography. Our solutions included comparing the 3D reconstruction CTA cone beam CT for arterial mapping (high contrast injection rate and contrast volume) in the common iliac artery, or using CO_2_ angiography. The low viscosity of CO_2_ could easily allow for injection by a microcatheter, and because CO_2_ floats within a vessel, it will go to the non-dependent site, so it will be relatively easy for the identification of the prostate arteryAnother benefit while using CO_2_ contrast is that both the antegrade and retrograde filling of the vessel could be save time for angiography. The overlap mode for viewing images of CO_2_ angiography was more effective. Final, using CO_2_ contrast would have minimal effect on renal function in the old patients. So, we changed our routine from using a microcatheter withiodine contrast injection, of which most of the contrast went to the gluteal arteries due to the limited injection rates.

Bilateral PAE might sometimes be difficult to achieve, especially in elderly patients with iliac/prostate artery stenosis and anatomical tortuosity [7]. Dr. Carnevale et al. also reported the technical aspects of PAE and the prognosis of unilateral PAE, where recurrence-free survival was found to be shorter than in bilateral PAE. The same finding was also reported in [8] Dr. Bilhim et al., in which the poor response rate was higher in unilateral PAE versus bilateral PAE (42% vs. 17.5%). In our clinical case reported here, there was a severe stenosis over the right prostate artery and the left prostate artery could support adequate blood flow towards the bilateral prostate glands via arterial anastomosis (Figure 2c,d). This finding suggested that we could evaluate the anatomy and the blood flow towards the prostate glands pre-operationally and start with the easy one first.

During the time of the present clinical case, the steerable microcatheter, or Rezum water vapor therapy, were not available in Taiwan [10]. Dr. Boeken et al. reported that type I anatomy (prostate artery and an inferior vesical artery originating from the anterior division of the internal iliac artery from a common trunk with the superior vesical artery) might require more microcatheters on average (1.15 ± 0.39 [SD]; range: 1–3) as the research team had changed to a smaller caliber microcatheter/steerable microcatheter for tightly angulated arterial take-off or tortuous arteries. Rezum water vapor therapy is another minimally invasive procedure for BPH treatment and could be performed under local anesthesia. It is recommended for men ≥50 years of age with a prostate volume between 30 cc and 80 cc. [11] Dr. Garden et al. reported compared outcomes after Rezum between men with small < 80 cc (SP) and large ≥ 80 cc prostates (LP); Rezum provided short-term symptomatic relief and improved voiding function comparable to SP patients. However, the mean days to foley removal (LP 9, SP 5.71, *p* = 0.003) and urosepsis rates (LP 5.56%, SP 0.00%, *p* = 0.002) differed.

A complicated urinary tract infection (UTI) is a symptomatic urinary infection accompanied by functional or structural abnormalities of the genitourinary tract. In male patients, BPH is a major cause of lower urinary tract obstruction, and bladder outlet obstruction can lead to UTI [12]. Recurrent or persistent UTI in men with BPH is an indication for surgical treatment. In our case, after PAE, no UTI occurred, nor other serious adverse events. The post-voiding residual volume improved from 155 mL to 87.6 mL and the patient did not require repeated admission nor other surgical treatments for his recurrent UTI.

During the PAE procedures, one thing we must note is the non-target embolization via the intraprostatic anastomosis connection to other organs. Micro-coil embolization, [13] Verapamil, or [14] micro-balloon catheter have been reported for non-target embolization prevention depending on the patient’s condition and anatomy. Local nitroglycerin injection before cone beam CT for the prostate artery is our hospital protocol because it is not only dilated in the fine terminal branches of the prostate artery, but it also possibly creates a temporary retrograde blood flow of the intraprostatic anastomosis connecting to other organs. Interactions with patients at this point are important for understanding the contrast-related warmness of the anal region and the penis. A higher dilution (up to 15 cc) of the microspheres with contrast allowed for confirming the site of embolization corresponding to the location; at the same time, it facilitated injection without the blockage of the microcatheter. In this patient, we failed the access to the left prostate artery, but there was an intraprostatic anastomosis connected to the terminal branch of both sides of the prostate arteries. Therefore, with unilateral microcatheter navigation, we completed bilateral embolization of his prostate arteries.

The TRA/dTRA approach for PAE had some advantages, especially when the patient could not stop anti-platelet/anticoagulation therapy, such as those undergoing post-coronary stenting, post-valve replacement, or were at high risk of stroke due to arrythmia. Due to the tortuous course of iliac arteries, some cases were relatively difficult when we approached the ipsilateral side from a femoral approach, and we even needed to puncture both femoral arteries. Also, the hemostasis was much easier and complications with hematomas or pseudoaneurysms were lower in the TRA/dTRA approach [15]. Patient satisfaction was also reported over the transfemoral approach. Immediate mobility was one of the key aspects. Currently, TRA/dTRA is my default approach for PAE procedures.

PAE for BPH could be repeated as needed [7]. Ideally, a benign disease should be treated with a minimally invasive procedure, especially in the elderly. Some doctors may perform coil embolization in the prostate artery with an assumption of no secondary PAE for the patient, but this is not always the case. Just like bronchial artery embolization (BAE) for hemoptysis, we should preserve the original feeder, and then make the repeat procedure possible and easier. If we sacrifice the bronchial artery, the repeat procedure will become much more difficult because the embolization the collaterals is not always feasible. Some authors discuss how the smaller size of the microspheres (100~300 µm) might experience more shrinkage in volume and probably undergo longer durations of being symptom- free but feature high rates of dysuria and penile ulcers compared with larger sized microspheres (300~500 µm). Despite the diameter of the microspheres for PAE procedures, small-sized or large-sized or mixed-sized microspheres all demonstrate the same clinical effectiveness [16].

## 5. Conclusions

In this case report, the treatment of a 100-year-old patient showed that PAE was a safe and efficient treatment for BPH, even though unilateral PAE was achieved. For elderly/non-surgical candidates, PAE is preferable to surgery, especially via a TRA/dTRA approach.

## Figures and Tables

**Table 1:** Baseline characteristics of patient and clinical outcomes after PAE.

	Baseline	1-Week	1-Month	6-Month	12-Month	24-Month
IPSS	23	15	8	7	5	5
Prostate Volume	89.39 cm^3^	NA	NA	NA	63.78 cm^3^	66.05 cm^3^
Post-void residual volume	155 mL	NA	NA	NA	87.6 mL	NA
Quality of Life	5	1	1	1	1	1
Complication	NA	0	0	0	0	0
Severe Complication	NA	0	0	0	0	0
Clinical Success	NA	Yes	Yes	Yes	Yes	Yes

## Data Availability

Data is contained within the article.
